# Relationship between sacroiliac joint involvement and intestinal disease activity in newly diagnosed Crohn’s disease

**DOI:** 10.1186/s12876-026-05122-8

**Published:** 2026-07-16

**Authors:** Gökhan Tonkaz, Hasan Hüseyin Can, Esma ÇINAR, Özgür Önal, Mehmet Tonkaz, Serdar Aslan, Duygu Erkal, Gökhan Aydın, Aykut Güruf, Ahmet Yasin Yitik

**Affiliations:** 1 Department of Radiology, Faculty of Medicine, Giresun University, Giresun, Turkey; 2 Department of Pathology, Faculty of Medicine, Giresun University, Giresun, Turkey; 3 Department of Public Health, Faculty of Medicine, Süleyman Demirel University, Isparta, Turkey; 4 Department of Gastroenterology, Faculty of Medicine, Giresun University, Giresun, Turkey; 5 Department of Biomedical Imaging and Image-guided Therapy, Medical University of Vienna, Vienna, Austria

**Keywords:** Inflammatory bowel disease, Sacroiliitis, Mayo endoscopic score, Geboes score, Magnetic resonance enterography, Pelvic computed tomography, Sacroiliac joint variants

## Abstract

**Background:**

Axial musculoskeletal involvement represents an important component among the extraintestinal manifestations of Crohn’s disease (CD). Sacroiliac joint (SIJ) involvement can be detected at an early stage using imaging modalities. The aim of this study was to evaluate the relationship between SIJ involvement and laboratory parameters, intestinal disease activity, and SIJ anatomical variations in patients with newly diagnosed CD.

**Methods:**

In this single-center retrospective imaging–pathology correlation study, 68 adult patients with a multidisciplinary diagnosis of Crohn’s disease established through clinical, endoscopic, radiological, and histopathological evaluation who underwent magnetic resonance enterography (MRE) and pelvic computed tomography (CT) examinations at the time of diagnosis were included. SIJ findings were classified as MRE-detected bone marrow edema-like findings, CT-detected structural SIJ abnormalities, or no involvement. SIJ anatomical forms were evaluated according to the morphological variation classification described by Ziegeler et al. Intestinal disease activity was analyzed using endoscopic (Mayo-based categories), histopathological (Geboes grading system), and clinical phenotype classification (Montreal classification) parameters.

**Results:**

MRE-detected bone marrow edema-like SIJ findings were detected in 9 patients (13.2%), CT-detected structural SIJ abnormalities in 9 patients (13.2%), and no SIJ involvement in 50 patients (73.5%). Platelet count, ferritin levels, and total iron-binding capacity (TIBC) differed significantly among the groups (*p* < 0.05). A statistically significant association was observed between endoscopic disease activity categories and SIJ findings (Fisher’s exact *p* < 0.001); however, the distribution pattern did not demonstrate a consistent increase in SIJ abnormalities with increasing endoscopic activity. In histopathological evaluation, a statistically significant association was found between SIJ involvement patterns and histopathological activity (Fisher’s exact *p* < 0.001); however, this association was primarily driven by the complete absence of severe histological activity in the CT-detected structural SIJ abnormalities group. In contrast, no significant relationship was found between disease location or behavior according to the Montreal classification and SIJ involvement patterns (*p* > 0.05). Similarly, no significant association was identified between SIJ anatomical variations and involvement patterns (*p* = 0.845).

**Conclusion:**

In patients with newly diagnosed CD, SIJ involvement appears to be most closely associated with histopathological disease activity. The association with endoscopic categories was statistically significant but showed an inverse, non-monotonic pattern, with SIJ findings clustering predominantly among patients with lower Mayo scores. In contrast, SIJ anatomical variations do not appear to play a determining role in the development of inflammatory or structural changes. These findings are exploratory in nature and indicate that prospective studies with clinical and rheumatologic correlation are required to better elucidate the relationship between intestinal and axial involvement in CD.

## Introduction

Crohn’s disease (CD) is a chronic inflammatory bowel disease (IBD) characterized by transmural inflammation that may involve any segment of the gastrointestinal tract. During the natural course of the disease, various extraintestinal manifestations may develop [1, 2]. Among these manifestations, musculoskeletal involvement constitutes a major component, with the axial form most frequently affecting the sacroiliac joint (SIJ) [3, 4].

In the literature, the reported prevalence of SIJ involvement in CD varies widely (2–46%), depending on the imaging modality used, diagnostic criteria applied, and characteristics of the studied population [5, 6]. SIJ involvement may present with clinical symptoms or remain clinically silent and is generally categorized as MRE-detected bone marrow edema-like findings and CT-detected structural SIJ abnormalities [7, 8]. Different imaging techniques offer complementary advantages in the evaluation of these changes. Computed tomography (CT) provides high diagnostic accuracy for detecting structural changes such as erosions, subchondral sclerosis, and ankylosis, and it also enables detailed assessment of SIJ anatomical forms [9]. Magnetic resonance imaging (MRI) is considered the principal modality for the early detection of active inflammatory findings, particularly bone marrow edema (BME) [10]. In the evaluation of intestinal involvement in CD, magnetic resonance enterography (MRE) has become a widely utilized imaging technique due to its absence of ionizing radiation and high diagnostic performance [11–13]. Although MRE is not specifically optimized for SIJ evaluation, the SIJs are typically included within the routine field of view, thereby allowing joint assessment without the need for additional imaging [14]. This provides a valuable opportunity to detect clinically unrecognized axial involvement, particularly in patients with newly diagnosed CD.

Evaluation of SIJ anatomical variants is an important factor in the interpretation of imaging findings. It has been suggested that anatomical variations of the SIJ may influence the distribution of mechanical stress across the joint and may predispose to the development of structural changes [15]. However, the relationship between these variations and SIJ involvement in patients with CD has been addressed in only a limited number of studies.

Assessment of disease activity in CD requires a multidimensional and integrated approach that extends beyond the evaluation of clinical symptoms alone. Within this framework, laboratory parameters, including acute phase reactants such as C-reactive protein (CRP) and erythrocyte sedimentation rate (ESR), provide indirect information regarding systemic inflammation; however, these markers do not always correlate with the severity of intestinal inflammation [16]. Endoscopic activity allows direct visual assessment of mucosal inflammation using Mayo-based categories as exploratory indicators of mucosal inflammatory burden [17]; whereas histopathological activity reflects microscopic inflammatory cell infiltration and epithelial damage through the Geboes grading system, playing a critical role in determining the true inflammatory burden, particularly in subclinical or endoscopically limited cases [18]. In addition, the Montreal classification, supported by imaging findings, standardizes the anatomical distribution and behavioral characteristics of the disease. Parameters such as disease location (with imaging playing a key role in the evaluation of small bowel involvement), age at onset, and disease behavior including penetrating or stricturing patterns are considered important determinants of clinical course and prognosis [19].

Although this multilayered evaluation system provides comprehensive information regarding intestinal disease activity and phenotypic characteristics, the relationship between these parameters and axial involvement, particularly SIJ changes, has not yet been sufficiently clarified in the literature [10]. In existing studies, the association between intestinal disease activity and SIJ findings in newly diagnosed CD has generally been examined in a fragmented manner; comprehensive analyses evaluating MRE-detected bone marrow edema-like findings and CT-detected structural SIJ abnormalities separately while also considering SIJ anatomical variants remain limited [20].

This study aimed to investigate, in patients with newly diagnosed CD, the relationship between SIJ anatomical variations, SIJ involvement, laboratory parameters, and endoscopic (Mayo-based categories), histopathological (Geboes grading system), and clinical phenotype (Montreal) findings using MRE and pelvic CT images obtained during routine clinical evaluation. By integrating the soft-tissue sensitivity of MRE with the superior capability of CT in evaluating osseous structures, this multimodal approach may contribute to a better understanding of axial involvement that may remain clinically silent in the early stages of CD and may help identify potential factors associated with this involvement.

## Methods

### Patients and study design

This study was designed as a single-center, retrospective, observational imaging–pathology correlation study. Archive records of the Radiology and Pathology Departments of Giresun University Training and Research Hospital were reviewed to identify adult patients who underwent endoscopic biopsy due to suspected IBD between October 2022 and December 2025 (*n* = 248).

The inclusion criteria were defined as follows: (i) multidisciplinary diagnosis of Crohn’s disease established through clinical, endoscopic, radiological, and histopathological evaluation, (ii) availability of MRE and pelvic CT examinations performed within ± 1 month of the diagnosis date, (iii) availability of an endoscopy report or recorded endoscopic images from the same time period, and (iv) accessibility of laboratory data. A total of 112 patients meeting these criteria were considered eligible for the study.

**Fig. 1:**
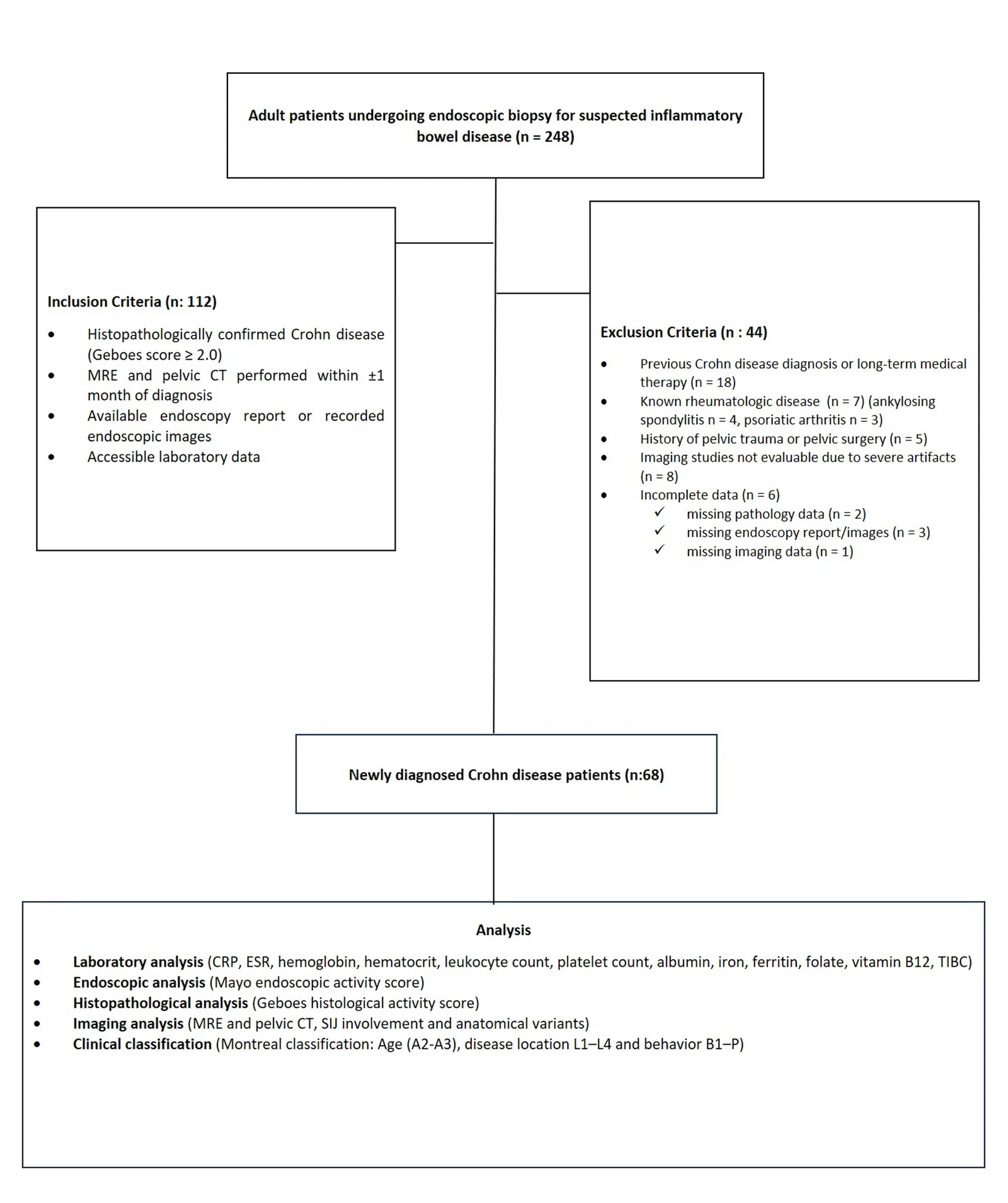
Flow chart of the study population showing the patient selection process

After reviewing the archive records, 44 patients were excluded for the following reasons: (i) prior diagnosis of CD or a history of long-term medical treatment (*n* = 18), (ii) presence of a known rheumatologic disease (ankylosing spondylitis *n* = 4, psoriatic arthritis *n* = 3) (*n* = 7), (iii) history of pelvic trauma (*n* = 3) or surgery (*n* = 2) (*n* = 5), (iv) imaging examinations that were non-evaluable due to significant artifacts (*n* = 8), and (v) incomplete data (missing pathology data *n* = 2, missing endoscopy report or images *n* = 3, missing imaging data *n* = 1) (*n* = 6). After applying the exclusion criteria, the remaining 68 patients were included in the study (Fig. 1).

In all included patients, intestinal disease activity was analyzed using endoscopic (Mayo-based categories) and histopathological (Geboes grading system) assessments, while disease location and behavior were determined according to the Montreal classification. SIJ evaluation was performed based on the presence of MRE-detected bone marrow edema-like findings and CT-detected structural SIJ abnormalities, and SIJ anatomical variations were additionally recorded using a morphological classification system described in the literature.

This study was conducted in accordance with the principles of the Declaration of Helsinki and was approved by the relevant local ethics committee (application no: BAEK-543, decision no: 07, date: 24.12.2025). Due to the retrospective nature of the study, the requirement for informed patient consent was waived by the ethics committee.

### Laboratory evaluation

Laboratory data of the patients included in the study were obtained from venous blood samples collected within one month prior to the endoscopic biopsy procedure on which the histopathological diagnosis was based and were retrospectively reviewed through the hospital information management system records. In patients with more than one laboratory measurement within the same time interval, the values closest to the biopsy date were included in the analyses.

The laboratory parameters evaluated were CRP, ESR, hemoglobin, hematocrit, leukocyte count, platelet count, albumin, serum iron level, ferritin, folate, vitamin B12, and total iron-binding capacity (TIBC). All laboratory analyses were performed in the central laboratory of our hospital using automated analyzers routinely employed in clinical practice, and the obtained results were recorded together with the corresponding reference ranges.

### Endoscopic evaluation

Endoscopic evaluations of the patients included in the study were retrospectively reviewed using colonoscopy reports and available endoscopic image records obtained as part of routine clinical practice at the time of CD diagnosis. Endoscopic examinations were performed by an experienced gastroenterologist, and the evaluations were analyzed in a standardized manner based on archived records.

**Fig. 2:**
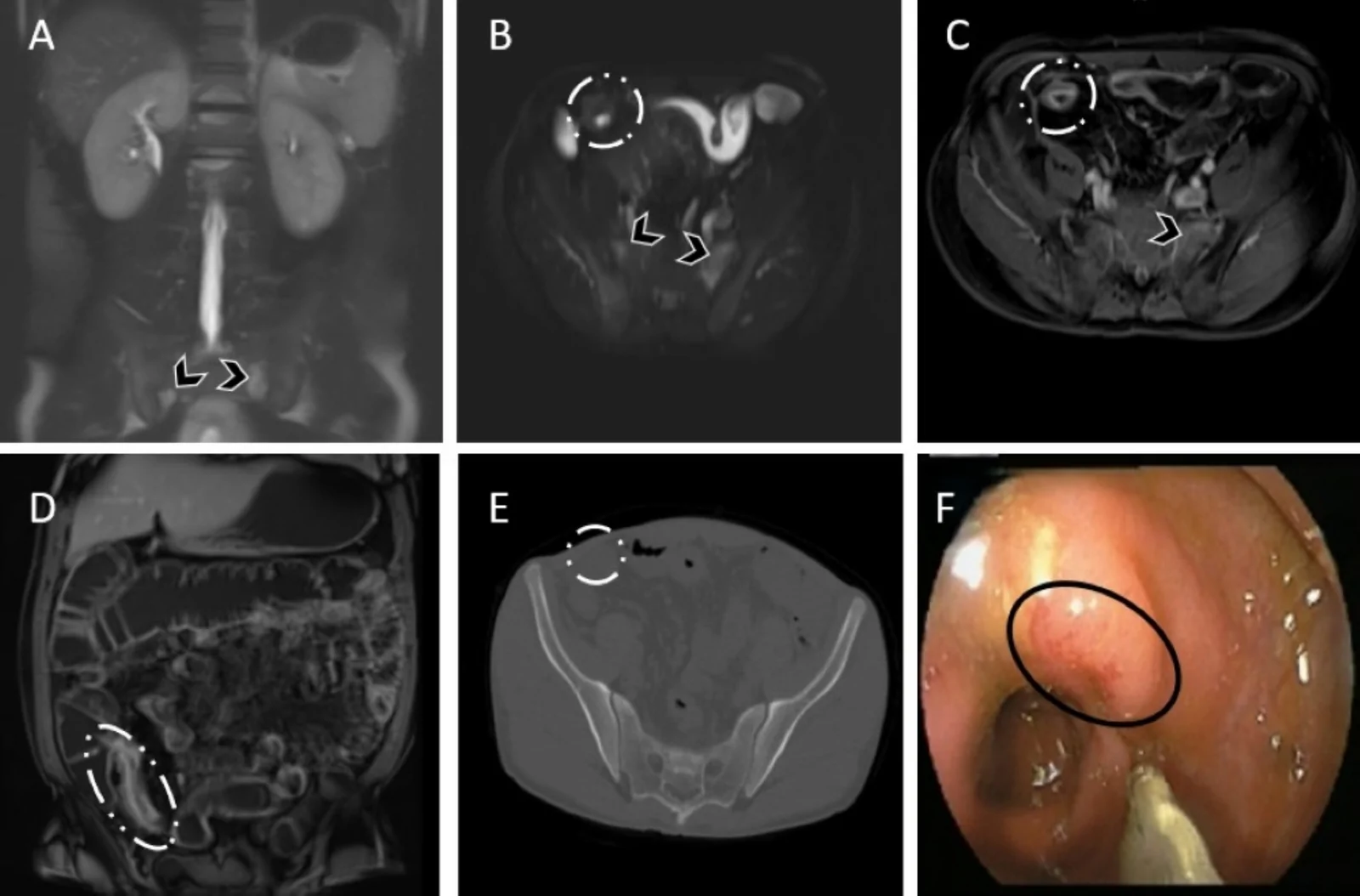
On HASTE fat-suppressed coronal (**A**) and axial (**B**) MRE images, bone marrow edema (arrowheads) reflecting active inflammatory changes in the sacrum at the RS1, RS2, LS1, and LS2 levels is observed, together with terminal ileitis (dotted circle). On post-contrast fat-suppressed axial (**C**) and coronal (**D**) T1-weighted images, terminal ileitis (dotted circle) and areas of contrast enhancement at the LS1 level consistent with active inflammatory changes (arrowhead) are demonstrated. On the axial pelvic CT image (**E**), terminal ileitis (dotted circle) is visible; however, no definite finding suggestive of SIJ involvement is identified. On the endoscopic image (**F**), mild erythema and decreased vascular pattern at the level of the ileocecal valve consistent with Mayo score 1 are observed (circle)

**Fig. 3:**
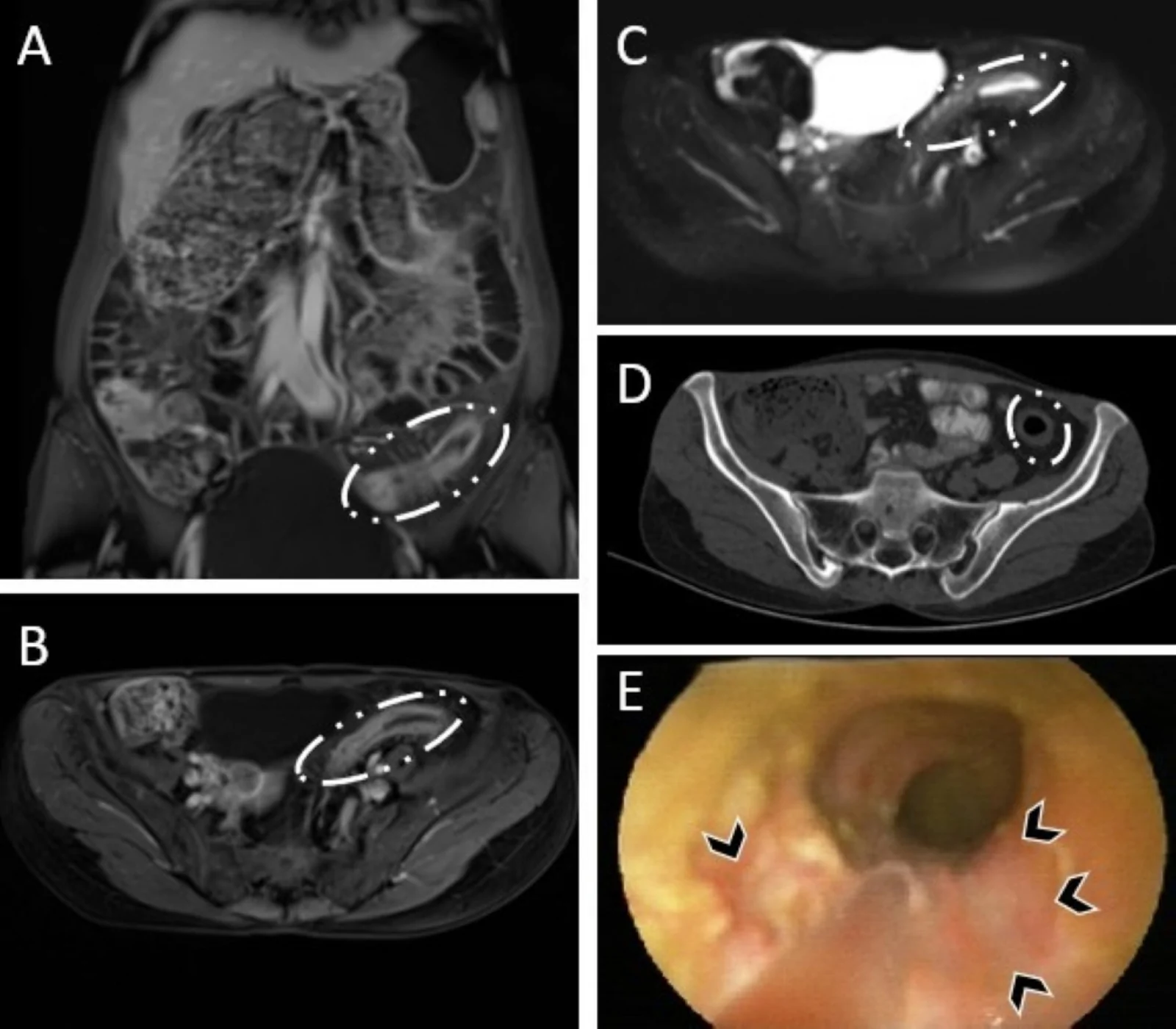
On post-contrast fat-suppressed coronal (**A**) and axial (**B**) T1-weighted images and HASTE fat-suppressed axial (**C**) MRE images, bowel wall thickening consistent with CD involvement is observed at the sigmoid and descending colon levels (dotted circle). On the axial pelvic CT image (**D**), intestinal disease involvement is visible (dotted circle); however, no definite finding suggestive of SIJ involvement is detected. In addition, the presence of bilateral accessory joint surfaces is consistent with a Type B anatomical variant. On the endoscopic image (**E**), spontaneous bleeding and diffuse ulceration areas along the colonic axis consistent with Mayo score 3 are observed (circle)

**Fig. 4:**
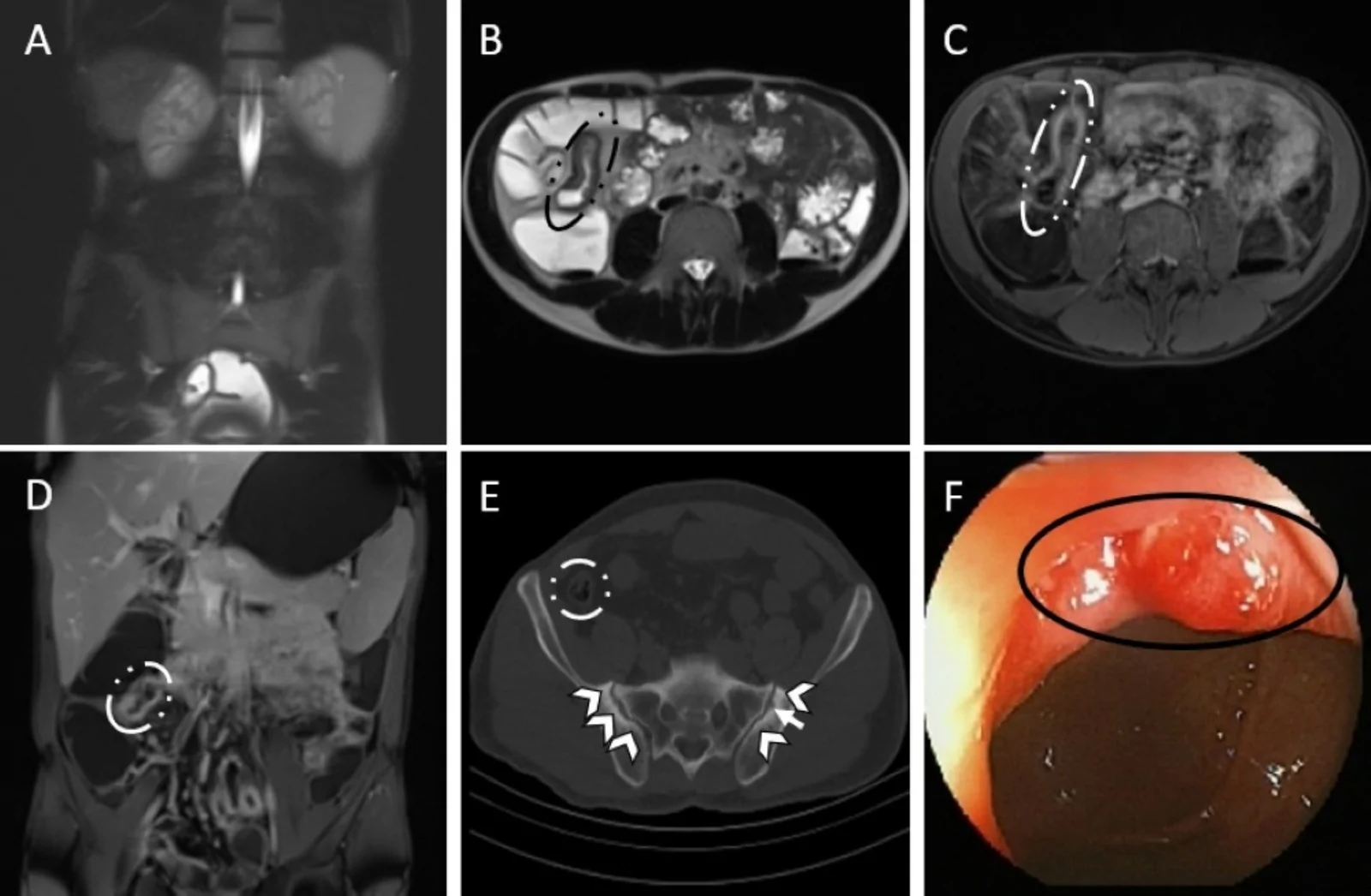
Terminal ileitis (dotted circle) is observed on HASTE non–fat-suppressed axial (**B**) images and on post-contrast fat-suppressed axial (**C**) and coronal (**D**) T1-weighted MRE images. On the HASTE fat-suppressed coronal (**A**) image, no definite finding suggestive of SIJ involvement is identified. On the axial pelvic CT image (**E**), terminal ileitis (dotted circle) is visible; in addition, bilateral iliac subchondral sclerosis (arrowheads) and erosion at the LI1 level (arrow), consistent with structural SIJ involvement, are observed. On the endoscopic image (**F**), marked erythema, erosions, and friability at the level of the ileocecal valve consistent with Mayo score 2 are observed (circle)

Because of the retrospective study design, Crohn’s disease-specific endoscopic activity indices such as SES-CD and CDEIS were not consistently available in archived records. Therefore, Mayo-based endoscopic categories documented in routine endoscopic reports were retrospectively used as exploratory indicators of mucosal inflammatory burden rather than validated measures of Crohn’s disease activity. Endoscopic disease activity was graded using the Mayo endoscopic score, a widely used scoring system in IBD [17]. According to this scoring system, a normal mucosal appearance with no evidence of inflammation was defined as score 0; mild erythema, decreased vascular pattern, and fine granularity as score 1 (Fig. 2F); marked erythema, loss of vascular pattern, erosions, and friability as score 2 (Fig. 4F); and spontaneous bleeding with diffuse ulceration as score 3 (Fig. 3E). Findings obtained from endoscopic examinations were analyzed independently of SIJ imaging results and other clinical data in order to minimize observer bias.

### Histopathological evaluation

Endoscopic biopsy specimens were processed through standard histopathological procedures, fixed in formalin, embedded in paraffin, and the obtained sections were stained with hematoxylin–eosin. All slides were retrospectively evaluated by a pathologist experienced in IBD, independently of clinical and imaging findings.

Crohn’s disease diagnosis was established through multidisciplinary evaluation integrating clinical presentation, endoscopic findings, radiological examinations, and histopathological assessment. Histopathological features supporting the diagnosis included chronic active inflammation, focal crypt architectural irregularity, segmental involvement pattern, transmural inflammatory features when assessable, and non-caseating granulomatous inflammation in appropriate cases. The degree of histopathological inflammatory activity was retrospectively categorized using the Geboes histological grading system available in archived pathology records. Although originally developed for ulcerative colitis, the Geboes system was used in the present study as an exploratory indicator of histological inflammatory burden rather than a Crohn’s disease-specific histological activity index, supported by expert consensus data suggesting its utility in Crohn’s disease research settings [18, 21, 22]. In this scoring system, structural changes, chronic inflammatory infiltration, the presence of neutrophils and eosinophils in the lamina propria, neutrophil infiltration into the epithelium, and crypt damage were evaluated under separate subcategories. Particular attention was paid to the severity of acute inflammation and the integrity of the epithelium. Histological activity was classified as follows: cases with a Geboes score between 2.0 and 3.0 were considered mild activity, those with a score between 3.1 and 4.3 as moderate activity, and those with a Geboes score ≥ 5.0 as severe activity (presence of erosion and/or ulceration) [21]. For statistical analysis, histopathological activity was categorized into two groups: mild–moderate activity and severe activity.

### Imaging evaluation

All radiological evaluations were performed independently by two radiologists with 6 and 8 years of professional experience in IBD and musculoskeletal imaging, who were blinded to the gastroenterological clinical data and histopathological findings. During the assessments, MRE and pelvic CT images were evaluated together. The SIJs were evaluated on routine MRE and CT examinations obtained primarily for intestinal assessment. Dedicated semi-coronal STIR/T2 fat-suppressed and semi-axial SIJ sequences recommended for dedicated sacroiliac joint assessment were not available, as imaging had not been performed specifically for rheumatologic evaluation. In the evaluations performed by both observers, findings related to SIJ involvement and intestinal disease characteristics were recorded separately. In cases where discrepancies occurred between the observers, a consensus was reached during a joint review session to establish the final decision.

#### MRE protocol

All MRE examinations were performed using a 1.5 Tesla MRI scanner (Magnetom Symphony, Siemens Medical Solutions, Erlangen, Germany, manufactured in 2016) located in the radiology unit of our hospital. Patients were evaluated after at least 6 h of fasting prior to the procedure, and to ensure adequate intestinal distension, they were instructed to drink approximately 2 L of a lactulose solution (Duphalac^®^) diluted with water 45 min before the examination. During the procedure, an intravenous antispasmodic agent (20 mg hyoscine butylbromide) was administered to reduce peristalsis and minimize motion artifacts. All patients were examined in the supine position.

The MRE protocol included standard sequences for the evaluation of small bowel morphology and inflammatory changes [23]. A steady-state free precession (SSFP) sequence was obtained in the coronal and axial planes to evaluate small bowel morphology, mesenteric structures, and vascularization. T2-weighted half-Fourier acquisition single-shot turbo spin-echo (HASTE) sequences were acquired in both coronal and axial planes with and without fat suppression and were used to assess bowel wall thickness, the presence of edema, ulcerations, and extraluminal fluid collections. Diffusion-weighted imaging (DWI) was obtained in the axial plane using a single-shot echo-planar imaging (EPI) technique (b values: 50, 400, and 800 s/mm²) to evaluate active inflammation. After intravenous administration of a gadolinium-based contrast agent (0.1 mmol/kg gadobutrol, Gadovist^®^), fat-suppressed T1-weighted images were acquired in the portal venous phase (60–70 s).

#### Pelvic CT protocol

All pelvic CT examinations were performed without contrast using a 128-slice multidetector CT scanner (Revolution EVO, GE Healthcare, Milwaukee, WI, USA, manufactured in 2020) located in the radiology unit of our hospital. Patients were scanned in the supine position with their arms raised above the head, from the end of inspiration in a craniocaudal direction. The scan range was planned to include the entire pelvis to allow complete evaluation of the SIJ.

The standard acquisition parameters were as follows: tube voltage 120 kV, automatic tube current modulation (100–450 mAs range, SMART mA), spiral pitch factor 0.984:1, collimation 64 × 0.625 mm, and gantry rotation time 0.6 s. All images were evaluated in the axial plane as well as in coronal and sagittal planes using multiplanar reconstruction images. Slice thickness and reconstruction interval were set at 1.25 mm to allow detailed analysis of osseous structures (e.g., subchondral sclerosis, erosion, ankylosis). During image reconstruction, iterative reconstruction algorithms (ASiR-V, GE Healthcare) were used to reduce radiation dose and improve image quality [24].

#### Evaluation of disease location and behavior according to the Montreal classification

In MRE examinations, intestinal disease involvement was evaluated according to the Montreal classification in terms of disease location and disease behavior [19]. In addition, the age at diagnosis of the patients was classified according to the Montreal classification; however, since the study included only adult patients, only the A2 (17–40 years) and A3 (> 40 years) groups were evaluated. Disease location was categorized as terminal ileum (L1), colon (L2), ileocolonic involvement (L3), and upper gastrointestinal tract involvement (L4). Disease behavior was classified based on MRE findings as inflammatory (B1), stricturing (B2), and penetrating (B3), while the presence of perianal disease was indicated using the Montreal classification modifier (p).

#### Sacroiliac joint evaluation

**Fig. 5:**
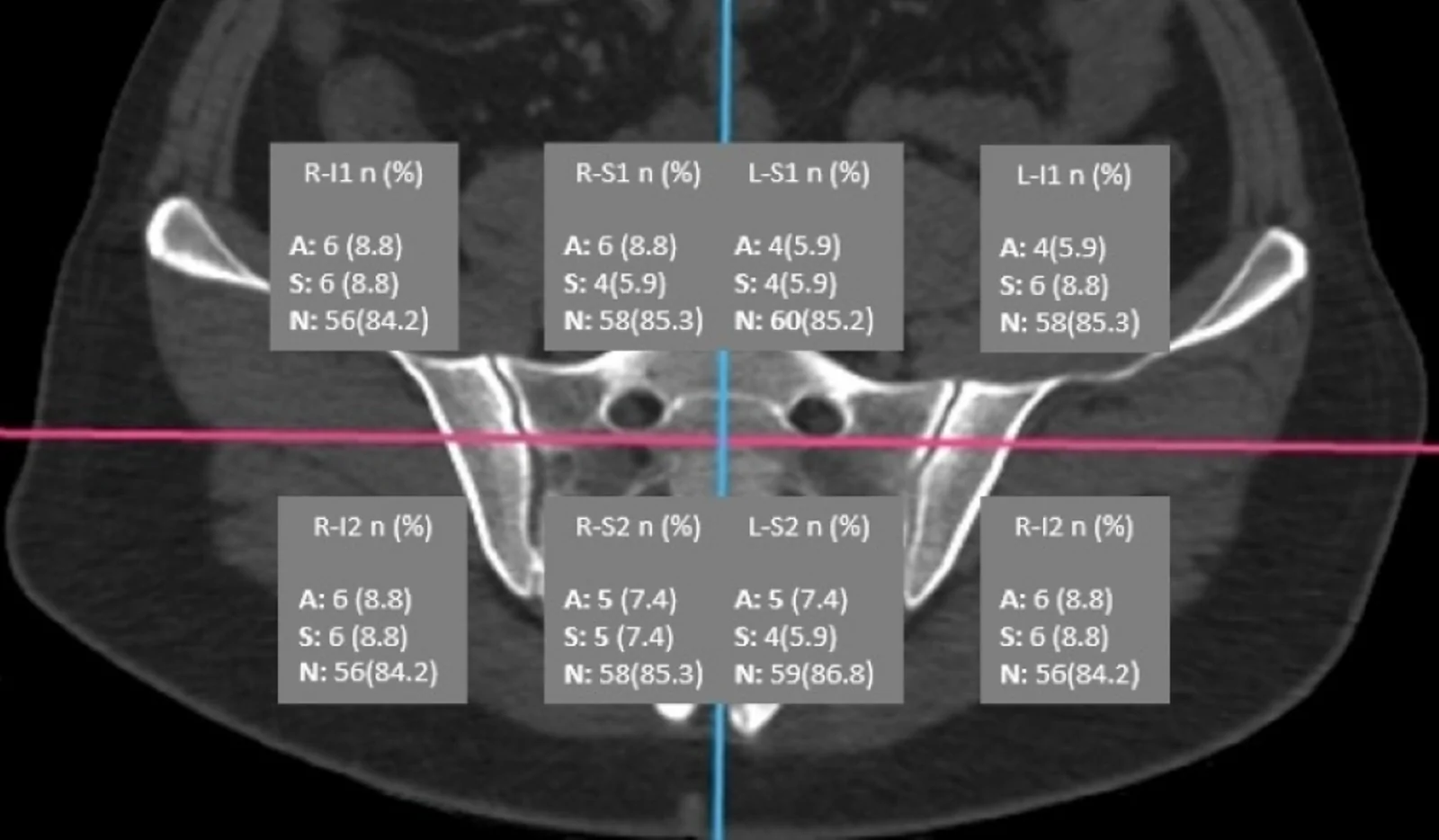
The SIJs are divided into eight anatomical subregions on pelvic CT images. Segmentation was performed using a horizontal line passing through the inferior margin of the first anterior sacral foramen on both sides (red) and a vertical line passing through the midline of the sacrum (blue). Accordingly, the regions were defined as right sacral upper (R-S1), right sacral lower (R-S2), left sacral upper (L-S1), left sacral lower (L-S2), right iliac upper (R-I1), right iliac lower (R-I2), left iliac upper (L-I1), and left iliac lower (L-I2). In each anatomical subregion, SIJ involvement was categorized as inflammatory (A), structural (S), or absent (N), and the results are presented as n (%)

The SIJs were analyzed by jointly evaluating the images obtained during MRE examinations and the available pelvic CT images. Both SIJs were examined segmentally, and each joint was divided into eight anatomical subregions based on the sacral segments and the corresponding regions of the ilium. The boundaries between the segments were determined using a horizontal line passing through the inferior margin of the first anterior sacral foramen on both sides and a vertical line passing through the joint space. These subregions were defined as right sacral upper (R-S1) and lower (R-S2), left sacral upper (L-S1) and lower (L-S2), right iliac upper (R-I1) and lower (R-I2), and left iliac upper (L-I1) and lower (L-I2) [20] (Fig. 5).

SIJ involvement was classified into three categories for descriptive purposes: MRE-detected bone marrow edema-like SIJ findings (referred to as ‘active inflammatory changes’ throughout this study), CT-detected structural SIJ abnormalities (referred to as ‘structural changes’), or no involvement. These terms are used descriptively and do not imply definitive diagnosis of sacroiliitis as defined by ASAS criteria. Active inflammatory changes were evaluated based on MRE findings, and the presence of BME (Fig. 2A–C, arrowheads), capsulitis, synovitis, and enthesitis was recorded. Structural changes were assessed using both MRE and CT findings and included subchondral sclerosis (Fig. 4E, arrowheads), erosion (Fig. 4E, arrow), fat metaplasia, ankylosis, and backfill [25]. In cases where both active inflammatory and structural changes were present, the lesions were classified as active inflammatory changes based on the principle that active inflammation leads to structural damage in the natural course of spondyloarthritis (SpA) [26]. Cases in which neither active inflammatory nor structural changes were observed were categorized as no involvement (Fig. 3B–D).

**Fig. 6:**
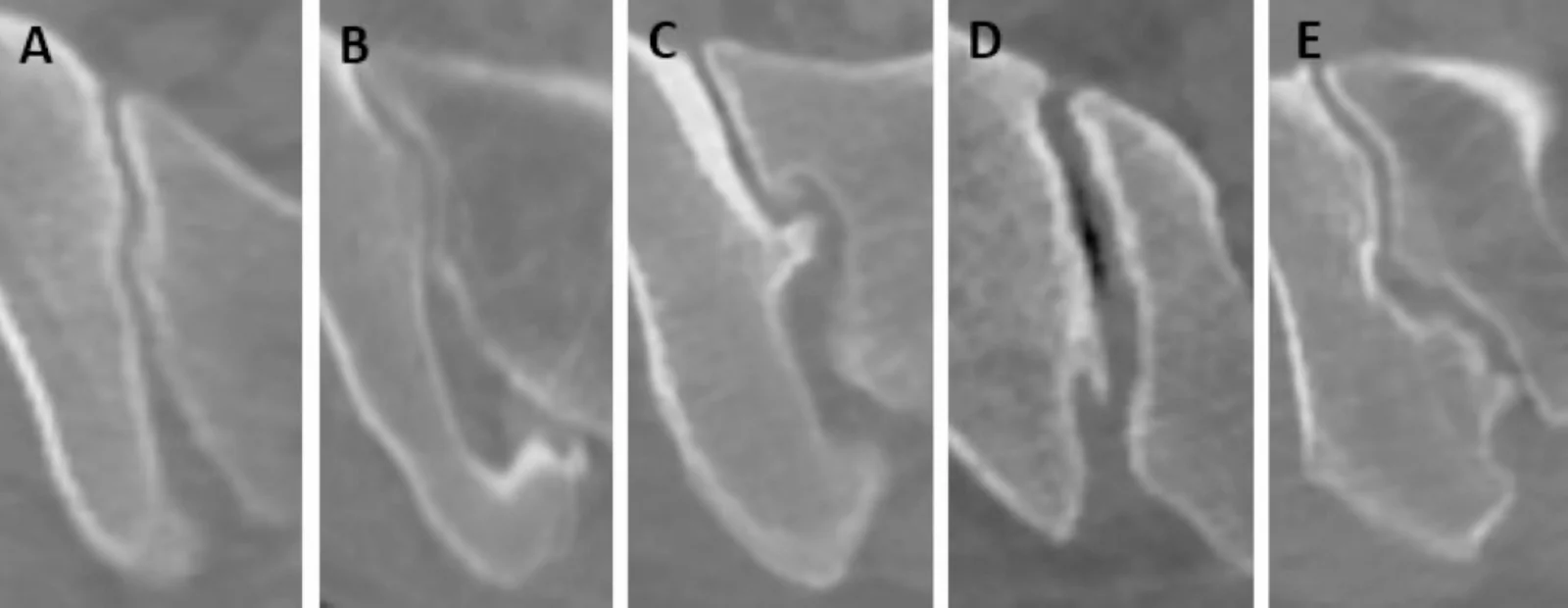
Anatomical variations of the SIJs. **A** Type A: classic crescent-shaped concave–convex joint surface; **B** Type B: presence of an accessory joint surface; **C** Type C: iliosacral complex (a prominent projection on the ilium with a corresponding concave surface on the sacrum); **D** Type D: bipartite ilium; **E** Type E: crescent-shaped ilium

The anatomical forms of the SIJs were evaluated according to the classification described by Ziegeler et al. [27]. In this classification, Type A represents the classic crescent-shaped concave–convex joint surface. Type B indicates the presence of an accessory joint surface, Type C the iliosacral complex (a prominent projection on the ilium with a corresponding concave surface on the sacrum), Type D bipartite ilium (failure of fusion of two separate iliac bone plates), Type E crescent-shaped ilium (concave iliac surface), and Type F semicircular defects or accessory ossification centers (Fig. 6).

### Statistical analysis

Statistical analyses were performed using IBM SPSS Statistics for Windows (version 30.0; IBM Corp., Armonk, NY, USA). The distribution characteristics of continuous variables were assessed using the Kolmogorov–Smirnov and Shapiro–Wilk tests. Continuous variables with normal distribution were presented as mean ± standard deviation, whereas non-normally distributed variables were expressed as median (minimum–maximum). Categorical variables were presented as numbers and percentages (%).

The relationships between disease behavior defined according to the Montreal classification (B1, B2, B3, p) and bowel segment involvement detected on MRE (L1–L4) were analyzed using the chi-square test for categorical variables; Fisher’s exact test was applied when the expected cell counts were insufficient. Similarly, the associations between SIJ involvement status (active inflammatory involvement, structural changes, no involvement) and clinical and radiological variables were evaluated using categorical analysis methods. In these analyses, effect sizes were reported using Cramer’s V. For variables with empty or sparse cells leading to quasi-complete separation (Mayo classification, Montreal age, Montreal behavior, Geboes classification, and SIJ anatomical type), Fisher’s exact test was used as the primary significance metric and effect size was not reported due to unreliable estimates.

Interobserver agreement for the evaluation of SIJ involvement patterns (active inflammatory changes, structural changes, no involvement) and anatomical form classification (Type A–F) was analyzed using Cohen’s kappa (κ) coefficient. Kappa coefficients were interpreted as follows: κ < 0.20 poor agreement, 0.21–0.40 fair agreement, 0.41–0.60 moderate agreement, 0.61–0.80 good agreement, and 0.81–1.00 very good agreement.

For comparisons of continuous variables between two groups, the independent samples t-test was used when the assumption of normal distribution was satisfied, and the Mann–Whitney U test was used when this assumption was not met. For comparisons among three or more groups, one-way analysis of variance (ANOVA) or the Kruskal–Wallis test was applied accordingly. When the Kruskal–Wallis test indicated statistical significance, pairwise comparisons were performed using Mann–Whitney U tests with Bonferroni correction. For Kruskal–Wallis analyses, effect size was calculated using the epsilon-squared (ε²) coefficient. In all statistical analyses, a p-value < 0.05 was considered statistically significant. The analyses performed in the present study should be considered exploratory because of the retrospective study design and limited subgroup sizes. The analyses were predefined according to the primary study objectives and focused on comparisons between sacroiliac joint involvement groups and clinical, laboratory, endoscopic, and histopathological parameters. No post-hoc or data-driven additional comparisons were performed. Formal multiple comparison correction was not applied due to the exploratory design and limited sample size, as such corrections could substantially increase the risk of type II error.

## Results

Among the 112 patients who met the inclusion criteria, 68 patients with CD were included in the study after the application of the exclusion criteria. Patients were divided into three groups according to SIJ findings: MRE-detected bone marrow edema-like SIJ findings (*n* = 9, 13.2%), CT-detected structural SIJ abnormalities (*n* = 9, 13.2%), and no SIJ involvement (*n* = 50, 73.5%). Evaluation of demographic characteristics showed no significant differences among the groups in terms of age, sex, or body mass index (*p* > 0.05). The demographic and clinical characteristics of the groups are summarized in Table 1.

**Table 1:** Distribution of Sacroiliac Joint Involvement Patterns Across Demographic and Clinical Variables

		Sacroiliac Joint Changes				
	Total	BME-positive	Structural	None	Fisher exact *p*	Cramer’s V
Sex Female Male	31 (45.6%) 37 (54.4%)	2 (22.2%) 7 (77.8%)	3 (33.3%) 6 (66.7%)	26 (52.0%) 24 (48.0%)	0.211	0.222
BMI < 18.5 (Underweight) 18.5–24.9 (Normal weight) 25–29.9 (Overweight) ≥ 30 (Obese)	9 (13.2%) 40 (58.8%) 11 (16.2%) 8 (11.8%)	2 (22.2%) 4 (44.4%) 1 (11.1%) 2 (22.2%)	1 (11.1%) 5 (55.6%) 2 (22.2%) 1 (11.1%)	6 (12.0%) 31 (62.0%) 8 (16.0%) 5 (10.0%)	0.824	0.187
Mayo Classification Level 1 Level 2 Level 3	16 (23.5%) 11 (16.2%) 41 (60.3%)	8 (88.9%) 1 (11.1%) 0 (0.0%)	8 (88.9%) 1 (11.1%) 0 (0.0%)	0 (0.0%) 9 (18.0%) 41 (82.0%)	**< 0.001**	-
Montreal Classification Age A2 A3	32 (47.1%) 36 (52.9%)	2 (22.2%) 7 (77.8%)	0 (0.0%) 9 (100.0%)	30 (60.0%) 20 (40.0%)	**< 0.001**	-
Montreal Classification Location L1 L2 L3 L4	33 (48.5%) 15 (22.1%) 13 (19.1%) 7 (10.3%)	4 (44.4%) 1 (11.1%) 2 (22.2%) 2 (22.2%)	3 (33.3%) 1 (11.1%) 4 (44.4%) 1 (11.1%)	26 (52.0%) 13 (26.0%) 7 (14.0%) 4 (8.0%)	0.277	0.230
Montreal Classification Behavior B1 B2 B3 P	55 (80.9%) 3 (4.4%) 2 (2.9%) 8 (11.8%)	6 (66.7%) 1 (11.1%) 0 (0.0%) 2 (22.2%)	7 (77.8%) 1 (11.1%) 0 (0.0%) 1 (11.1%)	42 (84.0%) 1 (2.0%) 2 (4.0%) 5 (10.0%)	0.374	-
Geboes Classification Severe Moderate–Mild	47 (69.1%) 21 (30.9%)	8 (88.9%) 1 (11.1%)	0 (0.0%) 9 (100.0%)	39 (78.0%) 11 (22.0%)	**< 0.001**	-
SIJ Anatomical Type A B C D E	44 (64.7%) 5 (7.4%) 4 (5.9%) 4 (5.9%) 11 (16.2%)	7 (77.8%) 0 (0.0%) 1 (11.1%) 0 (0.0%) 1 (11.1%)	6 (66.7%) 1 (11.1%) 1 (11.1%) 0 (0.0%) 1 (11.1%)	31 (62.0%) 4 (8.0%) 2 (4.0%) 4 (8.0%) 9 (18.0%)	0.866	-
	68 (100%)	9 (100%)	9 (100%)	50 (100%)		

Cramer’s V indicates effect size for categorical associations; effect size was not reported for Mayo Classification, Montreal Classification–Age, Montreal Classification–Behavior, Geboes Classification, and SIJ Anatomical Type due to empty cells leading to quasi-complete separation and unreliable estimates

BME-positive: MRE-detected bone marrow edema-like SIJ findings; Structural: CT-detected structural SIJ abnormalities

All *p*-values are based on Fisher’s exact test

Bold values indicate statistical significance (*p* < 0.05)

Among the laboratory parameters, platelet count, ferritin, and TIBC levels differed significantly among the groups (*p* = 0.027, ε²=0.061; *p* = 0.006, ε²=0.070; *p* = 0.044, ε²=0.039, respectively). In post-hoc analyses (Bonferroni-corrected Mann–Whitney U tests), platelet count and TIBC levels were significantly lower in the CT-detected structural SIJ abnormalities group compared with the group without SIJ involvement. Ferritin levels were significantly higher in the CT-detected structural SIJ abnormalities group compared with both the MRE-detected bone marrow edema-like SIJ findings group and the group without SIJ involvement. No significant differences were observed among the groups with respect to CRP, ESR, hemoglobin, hematocrit, leukocyte count, albumin, serum iron, folate, or vitamin B12 levels (*p* > 0.05). All laboratory findings are presented in Table 2.

**Table 2:** Distribution of Age and Laboratory Biomarkers Across Sacroiliac Joint Involvement Groups

Variable	SIJ Changes	*N*	Mean	SD	Min	Max	*p*	H Statistic	Epsilon square (ε^2^)
Age (years)	BME-positive	9	35.56	9.70	19	47	0.813	0.119	0.002
	Structural	9	34.89	11.57	24	54			
	None	50	37.18	9.71	20	55			
C-reactive protein (mg/L)	BME-positive	9	22.67	27.27	0	87	0.130	3.430	0.051
	Structural	9	13.22	16.25	0	40			
	None	50	10.98	26.97	0	186			
Erythrocyte sedimentation rate (mm/hour)	BME-positive	9	23.67	7.12	10	30	0.939	0.847	0.013
	Structural	9	27.89	17.76	5	60			
	None	50	25.68	16.54	3	75			
Hemoglobin (g/dL)	BME-positive	9	12.44	2.35	8	15	0.244	3.457	0.052
	Structural	9	13.44	2.07	10	16			
	None	50	12.14	2.18	7	16			
Hematocrit (%)	BME-positive	9	39.44	6.25	28	47	0.210	3.731	0.056
	Structural	9	41.89	3.52	37	47			
	None	50	37.92	6.39	23	49			
White blood cell count (×10³/µL)	BME-positive	9	7.44	3.01	3	12	0.857	0.652	0.010
	Structural	9	7.78	0.97	7	9			
	None	50	7.77	2.63	3	16			
Platelet count (×10³/µL)	BME-positive	9	314.67	67.68	204	431	**0.027**	4.098	**0.061**
	Structural	9	250.67	42.82	193	314			
	None	50	328.64	96.44	146	721			
Albumin (g/dL)	BME-positive	9	4.18	0.72	3	5	0.335	2.932	0.044
	Structural	9	4.06	0.63	3	5			
	None	50	4.34	0.59	3	6			
Iron (µg/dL)	BME-positive	9	58.22	20.47	28	90	0.266	3.313	0.049
	Structural	9	73.67	32.21	50	150			
	None	50	56.26	24.29	9	110			
Ferritin (ng/mL)	BME-positive	9	103.89	96.74	20	320	**0.006**	4.706	**0.070**
	Structural	9	231.11	81.98	120	350			
	None	50	110.66	99.73	3	385			
Total iron-binding capacity (µg/dL)	BME-positive	9	213.44	75.22	127	340	**0.044**	2.644	**0.039**
	Structural	9	154.00	43.88	92	211			
	None	50	213.74	70.73	120	450			
Vitamin B12 (pg/mL)	BME-positive	9	386.33	184.37	187	698	0.070	5.152	0.077
	Structural	9	346.22	158.95	135	691			
	None	50	277.96	133.22	90	733			
Folate (ng/mL)	BME-positive	9	5.11	2.26	2	9	0.419	3.017	0.045
	Structural	9	4.56	1.74	2	7			
	None	50	5.60	2.24	2	12			

Bold values indicate statistical significance (*p* < 0.05)

BME-positive: MRE-detected bone marrow edema-like SIJ findings; Structural: CT-detected structural SIJ abnormalities

When endoscopic activity was evaluated according to the Mayo score, a statistically significant association was found between SIJ involvement patterns and Mayo score categories (Fisher’s exact *p* < 0.001); however, the distribution pattern did not indicate a straightforward positive relationship between increasing intestinal inflammatory burden and SIJ abnormalities. In the MRE-detected bone marrow edema-like SIJ findings groups, Mayo score Level 1 cases predominated (88.9%), whereas in the group without SIJ involvement, Mayo score Level 3 cases constituted the majority (82.0%). The distribution of endoscopic findings according to the groups is presented in Table 1.

A significant association was found between the age parameter (A) of the Montreal classification and SIJ involvement status (Fisher’s exact *p* < 0.001). All patients in the CT-detected structural SIJ abnormalities group (100%) belonged to the A3 age group (> 40 years), and the proportion of A3 was also high in MRE-detected bone marrow edema-like SIJ findings group (77.8%). No significant association was observed between disease location (L1–L4) or disease behavior (B1–p) and SIJ involvement patterns (*p* > 0.05). Disease characteristics according to the Montreal classification are summarized in Table 1.

When SIJ anatomical forms were evaluated, Type A was the most common form in all groups, observed in 77.8% MRE-detected bone marrow edema-like SIJ findings group, 66.7% of the CT-detected structural SIJ abnormalities group, and 62.0% of the group without involvement. Type E was the second most frequent form and was detected more frequently in the group without involvement (18.0%) compared with the MRE-detected bone marrow edema-like SIJ findings and CT-detected structural SIJ abnormalities groups (11.1%). Type D was observed only in the group without involvement (8.0%). Type F anatomical form was not identified in any patient in the present study. No statistically significant association was found between SIJ anatomical type and involvement patterns (*p* = 0.845). The distribution of anatomical forms according to the groups is presented in Table 1.

When SIJ involvement was evaluated segmentally, the rates of involvement were low in all anatomical subregions, and MRE-detected bone marrow edema-like SIJ findings and CT-detected structural SIJ abnormalities showed similar distributions across the groups. In the segmental evaluation, the reported proportions (no involvement 84.6%; MRE-detected bone marrow edema-like findings 7.7%; CT-detected structural abnormalities 7.7%) refer to subregional involvement rates across all assessed joint subregions, not to patient-level prevalence (which was 73.5%, 13.2%, and 13.2%, respectively). No significant association was found between segmental distribution and involvement patterns (*p* = 1.00). The segmental distribution of involvement is shown in Fig. 5.

When histopathological activity was evaluated according to the Geboes score, a statistically significant association was found between SIJ involvement patterns and histopathological activity (Fisher’s exact *p* < 0.001). Severe histopathological activity (Geboes ≥ 5.0) was similarly prevalent in the MRE-detected bone marrow edema-like SIJ findings group (88.9%) and the no-involvement group (78.0%), indicating that these two groups were not meaningfully distinguished by histological severity. In contrast, all cases in the CT-detected structural SIJ abnormalities group (100%) were classified within the mild–moderate activity category. Accordingly, the significant association was primarily driven by the structural group’s complete absence of severe histological activity. The distribution of histopathological findings according to the groups is presented in Table 1.

Interobserver agreement for SIJ evaluations was high. The agreement rate for the presence of MRE-detected bone marrow edema-like SIJ findings was 95.6% (κ = 0.817; *p* < 0.001), for CT-detected structural SIJ abnormalities 94.1% (κ = 0.744; *p* < 0.001), and for cases without involvement 89.7% (κ = 0.740; *p* < 0.001). High interobserver agreement was also observed for the evaluation of SIJ anatomical forms (κ = 0.840; *p* < 0.001).

## Discussion

In this retrospective imaging–pathology correlation study, SIJ involvement patterns in newly diagnosed CD were evaluated multidimensionally in relation to endoscopic (Mayo-based categories), histopathological (Geboes grading system), and clinical phenotype (Montreal classification) activity indicators, laboratory parameters, and SIJ anatomical forms. Our findings demonstrate that SIJ involvement is most closely associated with histopathological disease activity. The relationship with endoscopic categories, while statistically significant, showed an inverse and non-monotonic pattern that warrants cautious interpretation.

Among the laboratory parameters, platelet count, ferritin, and TIBC levels differed significantly according to SIJ involvement patterns. Ferritin levels were significantly higher in CT-detected structural SIJ abnormalities group compared with both the MRE-detected bone marrow edema-like SIJ findings group and the group without involvement, whereas platelet count and TIBC levels were significantly lower compared with the group without SIJ involvement. Ferritin is not only an iron storage protein but also an acute-phase reactant that is known to increase during chronic inflammatory processes [28, 29]. However, within the spectrum of axial SpA, the relationship between structural damage and systemic inflammatory markers may be weak or discordant, and radiographic progression may occur independently of CRP levels [30, 31]. Disturbances in iron metabolism are common in IBD, and during chronic inflammation elevated hepcidin levels may lead to increased ferritin levels accompanied by decreased TIBC [32, 33]. In our study, the pattern of high ferritin and low TIBC observed in association with CT-detected structural SIJ abnormalities may reflect chronic inflammatory burden and prolonged immune activation rather than an active inflammatory phase. In contrast, the absence of differences in CRP and ESR levels among the groups supports the notion that axial involvement does not always parallel the systemic acute-phase response [30, 34]. The observed differences in ferritin and TIBC levels should be interpreted cautiously given the small subgroup sizes, and these exploratory findings may generate hypotheses for future prospective studies.

No significant association was observed between disease location (L1–L4) according to the Montreal classification and SIJ involvement patterns. In the literature, conflicting results have been reported regarding the relationship between disease location and the presence of sacroiliitis in CD. While some studies have suggested that colonic involvement may be more frequently associated with axial arthritis [35], larger cohort studies have failed to demonstrate a significant correlation between disease localization and axial involvement [10, 36]. Particularly in MRI-based studies, clinically silent sacroiliitis has been reported to develop independently of the anatomical distribution of intestinal disease [6, 37]. The findings of our study are consistent with previous reports showing no significant association between disease location and SIJ involvement patterns [10, 36, 38]. This observation suggests that sacroiliac involvement may be more closely related to shared immunopathogenetic mechanisms, and systemic inflammatory responses rather than the anatomical localization of intestinal inflammation. Therefore, axial involvement may be considered an independent extraintestinal manifestation rather than a phenotype directly reflecting the anatomical extent of CD. However, given the absence of rheumatologic correlation data — including HLA-B27 status, inflammatory back pain, peripheral arthritis, enthesitis, and family history — caution is warranted in drawing mechanistic conclusions. The present findings should not be interpreted as evidence of axial spondyloarthritis but rather as imaging-based observations requiring clinical correlation in future prospective studies.

When the effect of age was evaluated, sacroiliac joint changes were observed more frequently in the older age group (A3) according to the Montreal classification. This finding suggests that axial involvement in newly diagnosed CD may be associated both with active inflammatory changes that become more detectable with increasing age and with structural changes that may develop over time as a consequence of inflammatory processes that may have started prior to diagnosis [30]. In MRE- and CT-based studies, higher rates of sacroiliac joint abnormalities have similarly been reported in older patient groups [6, 36, 37]. The more frequent SIJ changes observed in the A3 group in our study support the possibility that age may represent an independent determinant of axial involvement; however, the interaction of this relationship with disease duration and the duration of subclinical inflammation requires clarification in prospective studies. Notably, all patients in the CT-detected structural SIJ abnormalities group belonged to the A3 (> 40 years) age category. Since erosion- and sclerosis-like changes on non-contrast CT are well documented in older individuals without spondyloarthritis, a degenerative or mechanical etiology cannot be excluded for a proportion — or the majority — of these findings.

In our study, the prevalence of MRE-detected bone marrow edema-like SIJ findings in the sacroiliac joint among patients with newly diagnosed CD was 13.2%. This rate is consistent with previous studies reporting variable prevalence rates of active sacroiliitis in CD, depending on the imaging modality and diagnostic criteria used [10, 14, 39]. The fact that MRE includes the SIJs within the field of view during routine intestinal evaluation provides an important advantage for detecting subclinical axial involvement that may remain clinically silent and may develop before rheumatologic symptoms appear [10, 14]. Nevertheless, the clinical significance of such incidentally detected findings remains uncertain in the absence of dedicated SIJ imaging sequences, HLA-B27 data, and rheumatologic assessment.

In the evaluation of SIJ anatomical forms, no statistically significant association was found between the defined morphological variations and SIJ involvement patterns. This finding suggests that anatomical variants alone are not determining factors in the development of inflammatory or structural changes. In the literature, SIJ variations have been reported to influence load transfer and biomechanical stress distribution, and certain configurations such as accessory SIJ or complex iliosacral morphology may be associated with mechanically induced BME [15, 27, 40]. However, imaging findings of mechanical BME and inflammatory BME may sometimes overlap on MRI, increasing the risk of false-positive interpretation of inflammatory sacroiliitis [40, 41]. Within the framework of the Assessment of SpondyloArthritis international Society (ASAS) criteria, it has been emphasized that for BME to be interpreted as inflammatory sacroiliitis, the typical subchondral localization of lesions, their distribution pattern, and accompanying structural changes should be evaluated together [41]. Therefore, the absence of an association between anatomical variants and inflammatory involvement in our study supports the notion that morphological configurations primarily reflect mechanical adaptation, whereas axial inflammation observed in CD likely results from multifactorial pathogenesis related to immunological and systemic inflammatory processes. Considering the roles of genetic predisposition, cytokine-mediated inflammation, and mucosal immune activation in axial involvement associated with IBD, the lack of a determining role for anatomical variations alone appears to be an expected finding.

The distribution of Mayo-based endoscopic categories across SIJ groups did not demonstrate a straightforward positive relationship between intestinal inflammatory burden and SIJ abnormalities. Although a statistically significant association was observed (Fisher’s exact *p* < 0.001), MRE-detected bone marrow edema-like SIJ findings and CT-detected structural SIJ abnormalities were more frequent in patients with lower Mayo scores. Effect size was not reported due to quasi-complete separation caused by empty cells. Several factors may contribute to this unexpected pattern: Mayo-based categories are not validated for CD; quasi-complete separation reduces the reliability of associations derived from this contingency table; and the limited subgroup sizes (*n* = 9 per group) reduce statistical power. From a biological standpoint, this discordance may further reflect the asynchronous course of musculoskeletal and intestinal manifestations and the cumulative nature of structural changes. Conversely, the presence of higher Mayo scores in cases without SIJ involvement supports the concept that the severity of mucosal inflammation does not necessarily correspond with axial involvement [42]. Although previous studies have reported weak or inconsistent associations between endoscopic activity and sacroiliitis [43, 44], some of these studies evaluated SIJ involvement only in terms of presence or absence without distinguishing between active and structural lesions. According to current imaging principles, however, the combined and detailed reporting of active and structural lesions is considered critical for accurate evaluation of sacroiliitis [45, 46]. It should also be noted that CT-detected structural SIJ abnormalities are not specific for axial spondyloarthritis and may reflect mechanical or degenerative processes, particularly in the absence of a control group and HLA-B27 data, as has been demonstrated in non-spondyloarthritis populations. In our study, analyzing SIJ changes as separate active and structural subgroups enabled a more refined evaluation of the relationship between endoscopic activity and axial involvement and provided a more detailed perspective on the heterogeneous nature of the intestinal–axial disease axis.

A statistically significant association was identified between histopathological activity and SIJ involvement patterns (Fisher’s exact *p* < 0.001). However, this association requires careful interpretation: severe histological activity was similarly prevalent in the BME-positive (88.9%) and no-involvement (78.0%) groups, indicating that these groups were not meaningfully distinguished by histological severity. The significant association was primarily driven by the structural group, which showed a complete absence of severe histological activity — consistent with the interpretation that CT-detected structural SIJ abnormalities may represent sequelae of prior inflammatory episodes rather than active ongoing inflammation. Indeed, extraintestinal manifestations may progress independently of intestinal disease activity [47, 48] and certain manifestations, particularly axial arthropathy, may follow a course independent of the clinical trajectory of CD [47]. Therefore, the absence of a consistent correlation between structural joint damage and current inflammatory activity further supports this interpretation; however, it remains exploratory given the cross-sectional design of the present study.

An important methodological consideration is the use of Mayo-based endoscopic categories and the Geboes histological grading system in a Crohn’s disease cohort. Although Crohn’s disease-specific indices such as SES-CD or CDEIS would have been preferable, these scores were not consistently available in archived records. Similarly, although the Geboes system was originally developed for ulcerative colitis, expert consensus studies have suggested its utility in Crohn’s disease research settings. Nevertheless, features such as patchy inflammation, transmural involvement, and granulomatous inflammation may not be fully captured by this system. Therefore, these findings should be interpreted as exploratory inflammatory burden indicators rather than validated disease activity measurements.

This study has several limitations. The retrospective design, the relatively limited sample size, and the use of data from a single center may restrict the generalizability of the results. The absence of dedicated semi-coronal STIR/T2 fat-suppressed and semi-axial SIJ sequences limits interpretation according to ASAS recommendations, and mild bone marrow edema-like changes may be subject to over- or under-interpretation without dedicated sequences. The absence of HLA-B27 status, rheumatologic examination findings, inflammatory back pain assessment, and other spondyloarthritis-related clinical data prevents definitive characterization of the imaging findings as axial spondyloarthritis-related sacroiliitis. A multivariable logistic regression analysis was explored; however, complete or near-complete separation between certain predictors and outcome groups precluded the construction of statistically reliable models. Information regarding symptom duration prior to diagnosis was not consistently available and could not be included in the analyses. Additionally, the presence of sparse and empty cells in key contingency tables introduces quasi-complete separation, rendering effect size estimates unreliable and raising the possibility that some statistically significant associations may represent chance findings. Nevertheless, the simultaneous multidimensional evaluation of histopathological, endoscopic, and radiological data within the same time interval represents one of the main strengths of the present study.

## Conclusions

In conclusion, this study demonstrates that SIJ involvement in newly diagnosed CD is most closely associated with histopathological disease activity. The association with endoscopic categories was statistically significant but showed an inverse, non-monotonic pattern, with SIJ findings clustering predominantly among patients with lower Mayo scores. In contrast, CT-detected structural SIJ abnormalities showed no correlation with current inflammatory activity and occurred exclusively in patients aged > 40 years, suggesting that these findings may most plausibly reflect degenerative or age-related changes rather than — or in addition to — inflammatory sequelae. Longitudinal conclusions cannot be drawn from the present cross-sectional data. These findings should be interpreted as exploratory observations. Our findings highlight the multidimensional and heterogeneous nature of the relationship between intestinal and axial involvement in CD and underscore the need for prospective studies with clinical and rheumatologic correlation, including HLA-B27 typing and dedicated SIJ imaging protocols, to better elucidate this axis.

Not applicable.

## Data Availability

The datasets used and/or analyzed during the current study are available from the corresponding author on reasonable request.

## Abbreviations

ANOVA: Analysis of variance
ASAS: Assessment of SpondyloArthritis international Society
BME: Bone marrow edema
CD: Crohn’s disease
CDEIS: Crohn’s Disease Endoscopic Index of Severity
CRP: C-reactive protein
CT: Computed tomography
DWI: Diffusion-weighted imaging
EPI: Echo-planar imaging
ESR: Erythrocyte sedimentation rate
HASTE: Half-Fourier acquisition single-shot turbo spin-echo
IBD: Inflammatory bowel disease
MRE: Magnetic resonance enterography
MRI: Magnetic resonance imaging
SES-CD: Simple Endoscopic Score for Crohn’s Disease
SIJ: Sacroiliac joint
SpA: Spondyloarthritis
SSFP: Steady-state free precession
TIBC: Total iron-binding capacity
